# Amino-Functionalized Metal–Organic Framework-Mediated Cellulose Aerogels for Efficient Cr(VI) Reduction

**DOI:** 10.3390/polym16223162

**Published:** 2024-11-13

**Authors:** Fan Yang, Dandan Hao, Miaomiao Wu, Bo Fu, Xiongfei Zhang

**Affiliations:** 1 College of Economics and Management, Nanjing Forestry University, Nanjing 210037, China; 2 College of Chemical Engineering, Jiangsu Co-Innovation Center of Efficient Processing and Utilization of Forest Resources, Nanjing Forestry University, Nanjing 210037, China; 3 School of Chemistry and Chemical Engineering, Southeast University, Nanjing 211189, China

**Keywords:** cellulose aerogel, Cr(VI) ions, MIL-125-NH_2_, photocatalysts

## Abstract

Industrialization activities have increased the discharge of wastewater that is polluted with hexavalent chromium (Cr(VI)), posing risks to ecosystems and humans. The photocatalytic reduction of Cr(VI) is viewed as a promising method for the removal of Cr(VI) species. However, developing photocatalysts with the desired catalytic activity, recyclability, and reusability remains a challenge. Herein, a composite aerogel was designed and fabricated with a Ti-based metal–organic framework (MIL-125-NH_2_) and carboxylated nanocellulose. MIL-125-NH_2_ presents a strong visible-light response, and the interactions between the amino groups of MIL-125-NH_2_ and the carboxyl groups of cellulose produce a strong interface affinity in the composites. The as-prepared aerogels exhibited a micro/macroporous structure. At an optimal MIL-125-NH_2_ loading of 55 wt%, the MC-5 sample showed a specific surface area of 582 m^2^·g^−1^. MC-5 achieved a photocatalytic Cr(VI) removal efficiency of 99.8%. Meanwhile, the aerogel-type photocatalysts demonstrated good stability and recycling ability, as MC-5 maintained a removal rate of 82% after 10 cycles. This work sheds light on the preparation of novel photocatalysts with three-dimensional structures for environmental remediation.

## 1. Introduction

Recently, water contaminated with heavy metal ions has emerged as a global challenge due to rapid industrialization and urbanization [1,2]. Among the various contaminants, hexavalent chromium (Cr(VI)) poses a severe threat to human health and the natural ecosystem owing to its toxicity, carcinogenicity, and persistence in the environment [3,4,5]. Traditional methods for Cr(VI) removal, such as chemical precipitation, ion exchange, and adsorption, often suffer from drawbacks like high cost, secondary pollution, and inefficiency [6,7]. In this context, photocatalysis has gained considerable attention as an innovative and promising technology for the remediation of Cr(VI)-contaminated water [8,9]. Photocatalysis leverages solar energy to activate semiconductor photocatalysts, which generate reactive species that are capable of reducing Cr(VI) to the less-toxic Cr(III) under mild conditions [10].

To date, various photocatalysts including metal oxides, g-C_3_N_4_ materials_,_ metal sulfides, and metal–organic frameworks (MOFs) have been developed for efficient Cr(VI) reduction [11,12,13]. In particular, MOFs have attracted widespread attention because of their high specific surface, desirable pore structure, easy functionality, and tunable bandgap [14,15]. The metal-oxo clusters in the MOF skeleton can serve as pollutant adsorption sites and catalytic active centers, as well as light-absorption units [4]. In previous studies, diverse kinds of MOFs (e.g., ZIF-8, MIL-53 (Fe), UiO-66-NH_2_, MIL-125-NH_2_) have been investigated for the photocatalytic removal of Cr(VI) under visible light [16]. Ti-based MOFs, which are constructed from Ti centers and organic substances, have demonstrated considerable Cr(VI) reduction potential due to their abundant titanium-oxo clusters [17]. Considering the facile functionalization of MOFs, group modification is a feasible strategy to enhance their photocatalytic performance [18,19]. For instance, Wang et al. prepared an MIL-125(Ti)-NH_2_ photocatalyst that was used for Cr(VI) reduction. They found that the introduction of amino groups enhanced the charge transfer process and considerably boosted the reduction efficiency [11].

However, MOF particles are commonly nano-sized; the difficulty of recycling and reusing MOFs have hindered practical applications of MOF-based photocatalysts [16,20]. The aggregation of powdered photocatalysts further reduces the exposure to active sites [21]. To solve these issues, the deposition of MOFs onto a three-dimensional and flexible substrate to obtain a composite photocatalyst seems to be a promising method [22,23,24]. Cellulose is an excellent substrate for MOFs due to being biodegradable and its sufficient surface hydroxyl/carboxyl groups [25,26]. For example, Ji and co-workers constructed a ZnO@ZIF-8(-NH_2_)/cellulose nanofiber foam with a Janus structure for removing tetracycline from wastewater [27]. Yang et al. immobilized MIL-125-NH_2_ particles onto cellulose membrane via an in situ growth method, which showed a 99% reduction efficiency for rhodamine B [28].

**Scheme 1:**
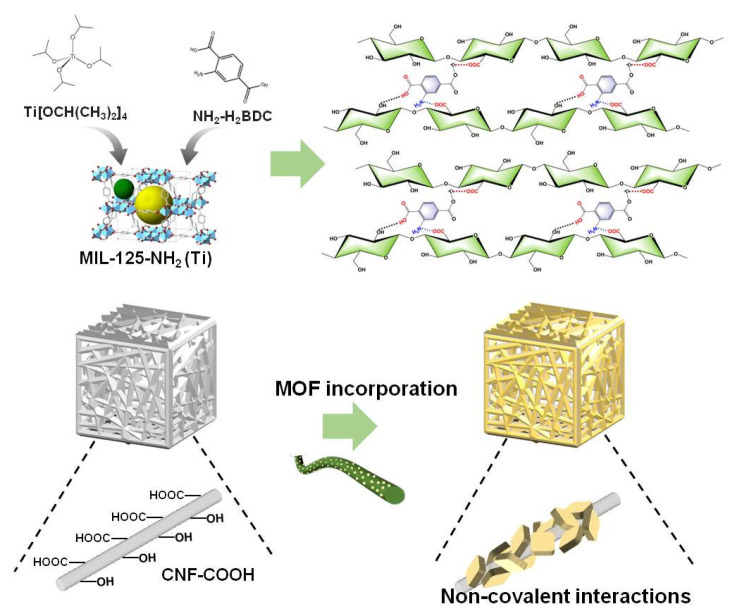
Schematic illustration of the fabrication process of the composite MIL-125-NH_2_/nanocellulose aerogels.

Herein, a porous aerogel was fabricated by combining MIL-125-NH_2_ and nanocellulose (Scheme 1). Amino-functionalized MIL-125-NH_2_ was selected based on its superb visible-light response. TEMPO-oxided nanocellulose, which has rich carboxyl groups in the C_6_ position of the glucose units, was used as a support to disperse and anchor the MOFs. We expected the formation of non-covalent interactions between the amino groups and carboxyl groups would promote the interface affinity. The obtained hybrid aerogels presented a highly porous structure, tight MOF attachment, and low density. The aerogels were utilized for the photocatalytic reduction of Cr(VI) under visible light. In addition, the recyclability and reusability of the aerogels were investigated. This work proposes a new strategy for the fabrication of a MOF-based aerogel photocatalyst.

## 2. Experimental Section

### 2.1. Materials

TEMPO-oxided nanocellulose suspension (CNFs, with a solid concentration of 1 wt%) was obtained from Mujingling Technology Co., Ltd., Tianjin, China. 2-Aminoterephthalic acid (BDC-NH_2_, 98%) and tetraisopropyl titanate (Ti(C_3_H_7_O)_4_, AR) were obtained from Shanghai Macklin Biochemical Co., Ltd., China. *N*′,*N*-dimethylformamide (DMF, 99.8%), and methanol were provided by Sinopharm Chemical Reagent Co., Ltd., Nanjing, China.

### 2.2. Synthesis of MIL-125-NH_2_

In brief, MIL-125-NH_2_ powders were synthesized through a hydrothermal procedure described in a previous paper with slight modifications [2,4]. First, 2.5 g of BDC-NH_2_ was dissolved in a mixed solution containing 33 mL of DMF and 8.3 mL of MeOH under ultrasonication. Second, 2.5 mL of Ti(C_3_H_7_O)_4_ was dropwise added into the mixed solution under continuous magnetic agitation for 30 min. Subsequently, the well-mixed solution was transferred into a sealed polytetrafluoroethylene vessel and heated at 150 °C for 16 h. After cooling to room temperature, the dark yellow precipitate was separated via centrifugation and thoroughly washed using DMF and MeOH. Finally, the obtained products were dried in a vacuum oven at 60 °C for 24 h.

### 2.3. Synthesis of Aerogels

In the synthesis, a certain mass of MIL-125-NH_2_ powder was uniformly dispersed into 19.1 mL of DI water with the aid of ultrasonication. Then, 0.4 g of CNF suspension was slowly poured into the MIL-125-NH_2_ suspension under continuous magnetic stirring. After being vigorously stirred for 6 h, the above mixture was injected into a square mold cavity (1 × 1 × 1 cm^3^) with a syringe and frozen in a refrigerator for 4 h. The hybrid aerogels were obtained after lyophilization. The amount of CNFs was fixed at 0.4 g throughout the preparation of the aerogels. According to the different mass values of MIL-125-NH_2_ (0.1 g, 0.2 g, 0.3 g, 0.4 g, 0.5 g, 0.6 g, 0.7 g, 0.8 g, 0.9 g), the composite samples were denoted as MC-x, where x refers to 1–9. For comparison, the pure CNF aerogel named C-A was produced following the same procedure without the addition of MIL-125-NH_2_.

### 2.4. Characterization

X-ray diffractometry (XRD) with Cu-Kα radiation was used to characterize the crystallinity and phase purity of the prepared MOF and aerogel samples (Ultima IV, Rigaku Corporation, Akishima, Japan). The morphological features of the photocatalysts were observed with field-emission scanning electron microscopy (FE-SEM, JSM-7600F, JEOL Corporation, Tokyo, Japan). Fourier-transform infrared (FT-IR) spectra were recorded on a VERTEX 80V spectrometer (Bruker Company, Karlsruhe, Germany) in a wavenumber range of 400–4000 cm^−1^. X-ray photoelectron spectroscopy (XPS) measurements were carried out on an AXISU ltraDLD instrument (Shimadzu Company, Kyoto, Japan). Zeta potential was assessed using a Zetasizer nano-ZS90 (Malvern, UK). Photoluminescence (PL) measurements were obtained with a Hitachi F-7000 spectrophotometer (Hitachi Company, Tokyo, Japan). The BET analysis was performed using BSD-PM2 equipment (Beishide Instrument, Beijing, China). The UV–vis diffuse reflectance spectra (DRS) were obtained on a UV-2450 spectrometer with BaSO_4_ as a reference (Shimadzu Company, Japan).

### 2.5. Photocatalytic Reduction of Cr(VI)

The photocatalytic tests of the MIL-125-NH_2_, C-A, and MIL-125-NH_2_/CNF composites were conducted under a 300 W xenon lamp (CEL-HXF300, λ > 420 nm, light intensity: 56 mW·cm^−2^) equipped with a 420 nm cut-off filter at ambient temperature. The pH value of the mixture was tuned to 2 with a diluted H_2_SO_4_ solution. In each experiment, 40 mg of photocatalyst was added to 10 mL of Cr(VI) aqueous solution at a concentration of 10 mg·L^−1^. Then, the solution was stirred in a dark environment for 1 h to achieve sorption equilibrium. Afterwards, the solution was placed in an irradiation reaction system with circulating cooling water passing throughout the whole process to maintain a constant temperature. During the reaction, 1.5 mL of solution was extracted with a syringe at a certain time intervals. The photocatalysts were removed via filtration through a 0.22 μm filter. The Cr(VI) concentration was determined by the diphenylcarbazide colorimetric approach with ultraviolet spectroscopy at 540 nm (the standard plot is provided in Figure S1, Supplementary Materials; the detailed measuring procedures are also provided in the Supplementary Materials) [18]. The removal efficiency of Cr(VI) was calculated with the following equation:

Removal efficiency (%) = (1 − C/C_0_)*100%

## 3. Results and Discussion

### 3.1. Synthesis

To prepare a composite aerogel, MIL-125-NH_2_ powders were presynthesized and added to a CNF suspension. CNF, characterized by a high aspect ratio, was selected as the starting material due to its ease of processing into aerogels that maintain complex entangled networks [21,29]. Specially, CNFs with abundant hydroxyl/carboxyl groups are beneficial for the attachment of MOF particles with functional amino groups through non-covalent interactions [29]. CNF exhibited a negative zeta potential of −17.9 mV. By contrast, MIL-125-NH_2_ had a positive charge of 59.2 mV (Figure S2, Supplementary Materials) because of the existence of unbalanced metal ions. The electrostatic interactions arising from the contrasting surface zeta potential values facilitated self-assembly between the cellulose substrate and the MOFs. This effective integration not only prevented the agglomeration of catalysts but also constructed supporting photocatalysts that inherited the 3D structure of the cellulose aerogels and the high activity of MIL-125-NH_2_.

**Figure 1:**
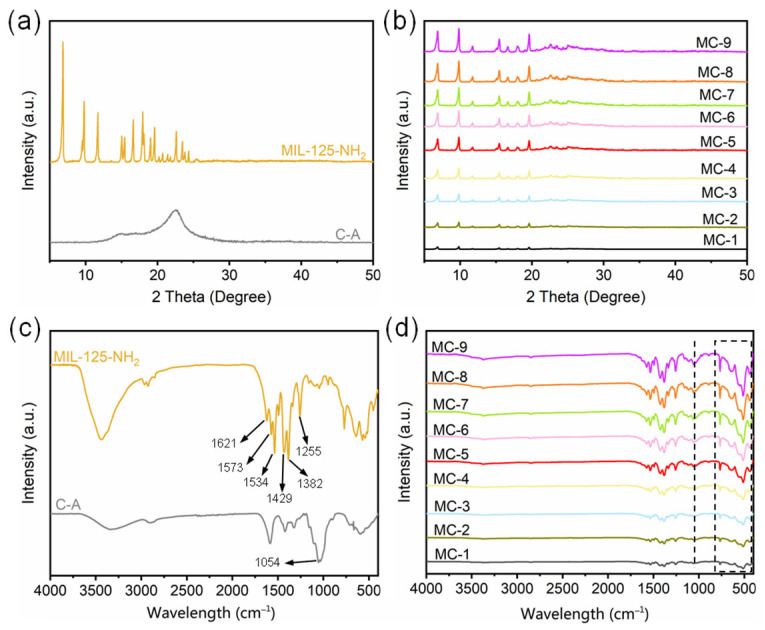
(**a**,**b**) XRD patterns and (**c**,**d**) FT-IR spectra of the C-A, MIL-125-NH_2_, and composite aerogels.

The phase structures of the synthesized samples were examined by XRD. As shown in Figure 1a, the strong diffraction peaks of MIL-125-NH_2_ at 6.8° (110), 9.8° (002), and 15.4° (222) align well with the CIF-file (CCDC 751157) [30]. The blank C-A aerogel exhibited a broad peak at 22.5°, corresponding to the (200) crystal face of type I cellulose [31]. The main reflections of MIL-125-NH_2_ were well preserved in the composite aerogels, and the characteristic peaks intensified with the increase in MOF content (Figure 1b). This indicated the successful integration of MIL-125-NH_2_ into the cellulose aerogel without affecting its crystal configuration [32]. In addition, the peaks attributed to C-A were weak, which could be explained by the low crystallinity of cellulose [33].

To further explore the group interactions between MIL-125-NH_2_ and the CNFs, the chemical structures of the prepared samples were analyzed by FT-IR. As displayed in in Figure 1c, the broad absorption band of MIL-125-NH_2_ at 3440 cm^−1^ was due to the dissociated solvent molecules trapped in the pores and the stretching vibration of the hydroxyl groups from adsorbed water [17]. The peak centered at 1255 cm^−1^ is ascribed to the stretching vibration of C-N. The bending vibration of C=O belonging the aromatic amine was observed at 1621 cm^−1^. The apparent absorption peaks located at 1429 cm^−1^ and 1534 cm^−1^ are associated with the asymmetric vibrations of carboxyl functional groups, while the absorption peaks at 1382 cm^−1^ and 1573 cm^−1^ are attributed to the symmetric vibrations of the carboxyl groups [34]. The absorption bands in the 400–800 cm^−1^ range correspond to the stretching vibration peaks of Ti-O groups [35]. With the increase in MOF content, the intensity of these characteristic peaks increased, confirming the successful preparation of the composite aerogels. For the FT-IR spectrum of the cellulose aerogel, C-A, a unique stretching vibration peak of C-O-C groups in the pyranose ring was observed at 1054 cm^−1^ [36]. As illustrated in Figure 1d, all composite aerogels exhibited spectra similar to those of the MIL-125-NH_2_ powder, indicating that the structure of MIL-125-NH_2_ was preserved during the self-assembly process. Additionally, the absorption peak around 3340 cm^−1^ corresponds to the stretching vibration of the -OH groups in CNF. Compared to pure MOFs and C-A, the characteristic peaks of the composite aerogel in the 3300–3400 cm^−1^ range were weaker and wider, suggesting interactions between CNF and MIL-125-NH_2_ [37].

**Figure 2:**
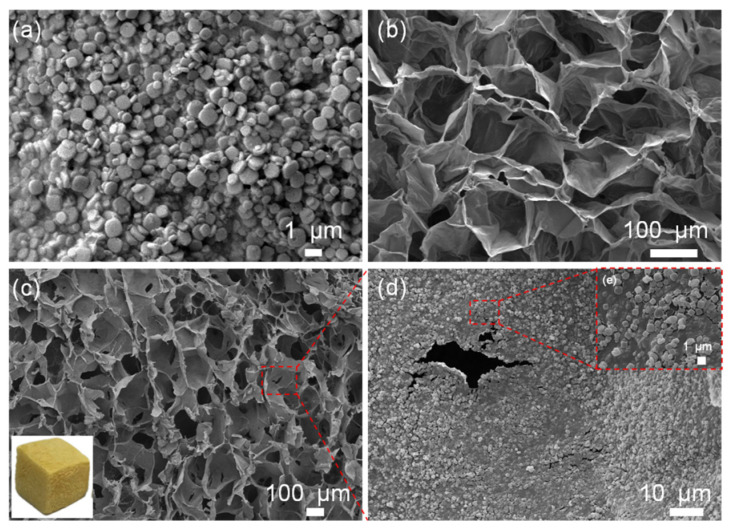
SEM images of (**a**) pristine MIL-125-NH_2_ powder, (**b**) C-A aerogel, (**c**,**d**) hybrid MC-5 aerogel, and (**e**) high-resolution view of MC-5.

The SEM technique was employed to examine the microstructures of the materials. Pure MIL-125-NH_2_ (Figure 2a) exhibited regular nanoplates with an average size of approximately 600 nm [17]. The agglomeration of MIL-125-NH_2_ powders was evident. The bare nanocellulose aerogel in Figure 2b displays a three-dimensional porous structure resulting from the freeze-drying process. This highly porous network facilitated mass transport and light scattering. However, the uniform micron-scale pores demonstrated loose and wrinkled characteristics, indicative of the softness and low mechanical strength of C-A aerogels. In contrast, the SEM images of MC-5 (Figure 2c) revealed a denser porous structure upon incorporation of MIL-125-NH_2_, likely due to the tight interactions between the components. Meanwhile, the physical photograph of the composite aerogel can be seen in the lower left corner in the figure, which had good elasticity, compression resistance, mechanical properties, and stability, which may be due to the incorporation of MIL-125-NH_2_ nanoparticles not only preventing the serious collapse of the aerogel structure but also contributing to the rapid recovery of the pore structure after compression. The magnified views of the composite aerogel (Figure 2d,e) showed that the aerogel matrix acted as a mechanical support, ensuring the uniform dispersion of MIL-125-NH_2_ nanocrystals and promoting contact between the target pollutants and the photocatalyst during the photocatalytic process.

**Figure 3:**
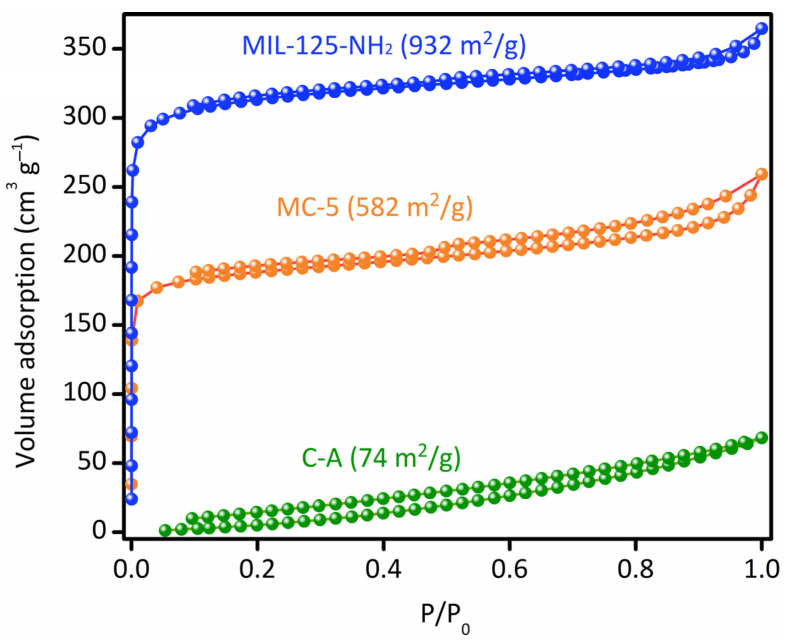
N_2_ sorption isotherms of the C-A, MIL-125-NH_2_, and composite MC-5 aerogels.

To ascertain the specific surface area and pore size distribution of the samples, N_2_ adsorption–desorption analyses were performed. As illustrated in Figure 3, pristine MIL-125-NH_2_ displayed a Type I adsorption isotherm according to the IUPAC classification, indicative of a typical microporous structure. Conversely, MC-5 exhibited a mixed I/IV isotherm, with a pronounced increase in the adsorption curve at P/P_0_ < 0.1, suggesting the effective retention of the microporous structure of MIL-125-NH_2_ within the aerogel. Notably, a distinct hysteresis loop was observed in the P/P_0_ range of 0.4 to 1, confirming the presence of mesopores. MIL-125-NH_2_ possessed a specific surface area of 932 m^2^·g^−1^, whereas the blank cellulose aerogel (C-A) exhibited a surface area of only 74 m^2^·g^−1^, consistent with its macroporous structure observed by SEM. The composite aerogel MC-5 showed a specific surface area of 582 m^2^·g^−1^, which was higher than that of the bare cellulose aerogel, demonstrating the wide dispersion of MIL-125-NH_2_ within the cellulose matrix. Moreover, MC-5 displayed a hierarchical porous structure, with macropores facilitating rapid pollutant diffusion and meso–micropores contributing to high porosity and exceptional adsorption capacity [31]. The large specific surface area and appropriate pore volume of the composite aerogel (Table S1, Supplementary Materials) not only provided abundant adsorption sites for target pollutants but also enhanced the capture of visible light and facilitated charge transport.

**Figure 4:**
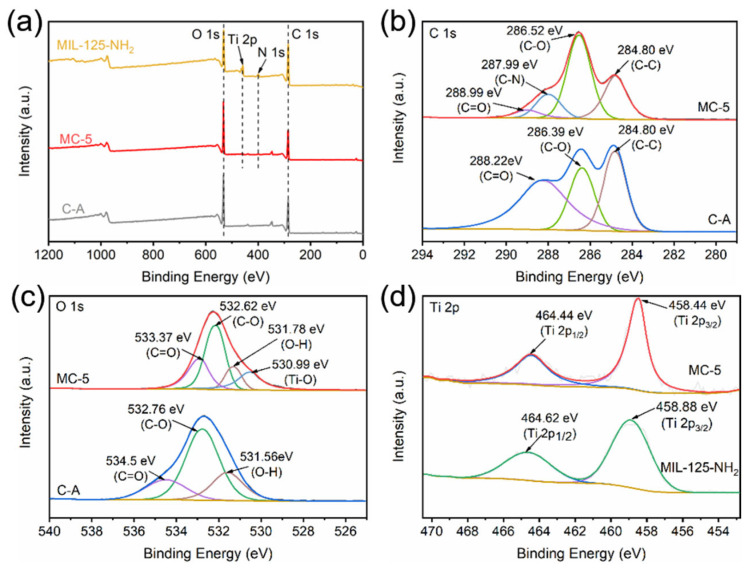
XPS spectra: (**a**) full survey of C-A, MIL-125-NH_2_, and MC-5 samples. High-resolution spectra for (**b**) C 1s, (**c**) O 1s, and (**d**) Ti 2p elements.

XPS was employed to elucidate the surface chemical compositions and states of the samples. The surveyed XPS spectrum (Figure 4a) revealed distinct peaks corresponding to C, N, O, and Ti in the hybrid aerogel. Close examination of the C 1s peak-splitting results (Figure 4b) demonstrated a redshift in the C-O and C=O peaks of the MC-5 composite aerogel compared to the C-A aerogel, indicating a change in the electron density around these groups in the cellulose molecular chain due to the incorporation of MIL-125-NH_2_ [38]. A similar shift in the binding energy of the O-H groups was observed in the high-resolution XPS spectrum of O 1s (Figure 4c). These observations suggested the presence of hydrogen bond interactions between cellulose and MIL-125-NH_2_. Figure 4d shows that the Ti 2p1/2 and Ti 2p3/2 peaks shifted to lower binding energies in the composite aerogel, likely due to group linkages between the metal center of MIL-125-NH_2_ and the carboxyl groups of cellulose [17,39]. The cellulose aerogel, with its abundant surface groups, provided robust anchoring sites for the MOFs, facilitating their tight immobilization and superior dispersion.

### 3.2. Photocatalytic Performance of Cr(VI) Reduction

**Figure 5:**
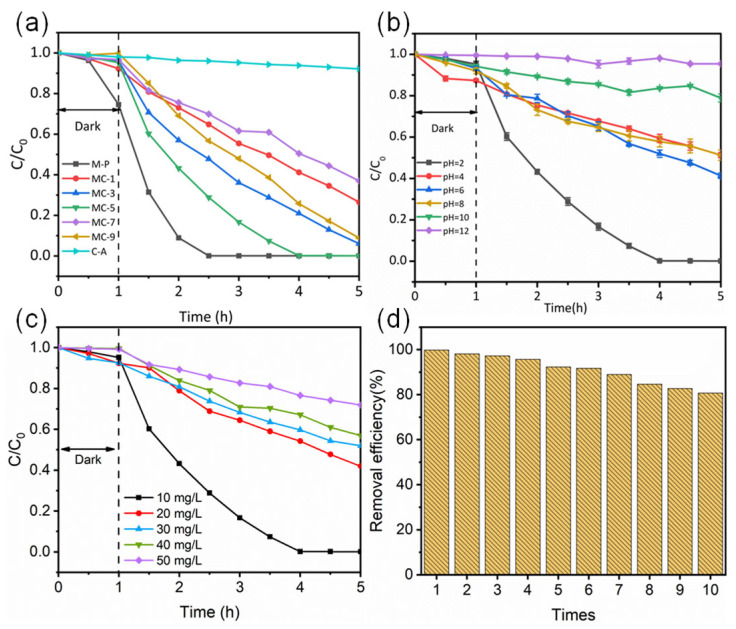
(**a**) Photocatalytic Cr(VI) reduction performance of different samples, (**b**) effects of pH on Cr(VI) reduction with MC-5, (**c**) effects of initial Cr(VI) concentration on Cr(VI) reduction, and (**d**) cyclic photocatalytic removal performance of Cr(VI) with MC-5.

The photocatalytic activity of the synthesized composites was assessed. Under a dark environment, all aerogels had poor Cr(VI) adsorption ability. Notably, as displayed in Figure 5a, the blank cellulose aerogel exhibited a Cr(VI) removal rate of approximately 10%. In contrast, the optimal composite sample, MC-5, achieved a remarkable reduction efficiency of 99.8%. However, the excessive loading of MIL-125-NH_2_ led to a decline in performance, attributed to factors such as agglomeration, uneven dispersion, and the formation of recombination centers. Despite this, the composite aerogel did not exhibit a substantial improvement compared with pure MIL-125-NH_2_, which might have been due to the low content of active photocatalyst within the composites and the inert nature of the cellulose aerogel. The ease of purification and extraction of these hybrid aerogels renders them highly suitable for practical applications. Furthermore, the composite aerogel in this study outperformed other reported MOF-based photocatalysts for Cr(VI) reduction [40]. Given its optimal photocatalytic performance, MC-5 was selected for the subsequent experiments.

To investigate the optimal photocatalytic conditions, the effects of pH (from 2 to 12) and initial pollutant concentration were examined. As depicted in Figure 5b, the highest Cr(VI) reduction efficiency with MC-5 was achieved at a pH of 2, with lower rates observed under alkaline conditions. This suggests that acidic conditions favor the photocatalytic reduction of Cr(VI) [41]. Additionally, as shown in Figure 5c, the removal efficiency negatively correlated with the initial Cr(VI) concentration, indicating that an excess of pollutants can overwhelm the available active sites, thus impeding removal efficiency. The composite aerogel materials in this study were also compared with those reported in the published literature for the removal of Cr(VI). Compared with the other photocatalysts, the as-prepared composite materials effectively removed Cr(VI) contamination from water (Table S2, Supplementary Materials).

Furthermore, the reusability of MC-5 during Cr(VI) reduction was assessed through repeated experiments conducted under identical conditions. As depicted in Figure 5d, MC-5 maintained a removal rate exceeding 82% even after ten cycles. Notably, the skeletal structure of the powdered photocatalysts, particularly MOFs, was prone to collapse under highly acidic conditions. To address this, we analyzed the reaction solution after photocatalysis using ICP and found that titanium was hard to detect, confirming the structural integrity of the composite aerogel throughout the photocatalytic process. Additionally, the XRD patterns and SEM images of the recycled aerogel (Figure S3, Supplementary Materials) revealed no significant changes in its crystal structure compared to the original sample, further corroborating the robust structure of the aerogel.

**Figure 6:**
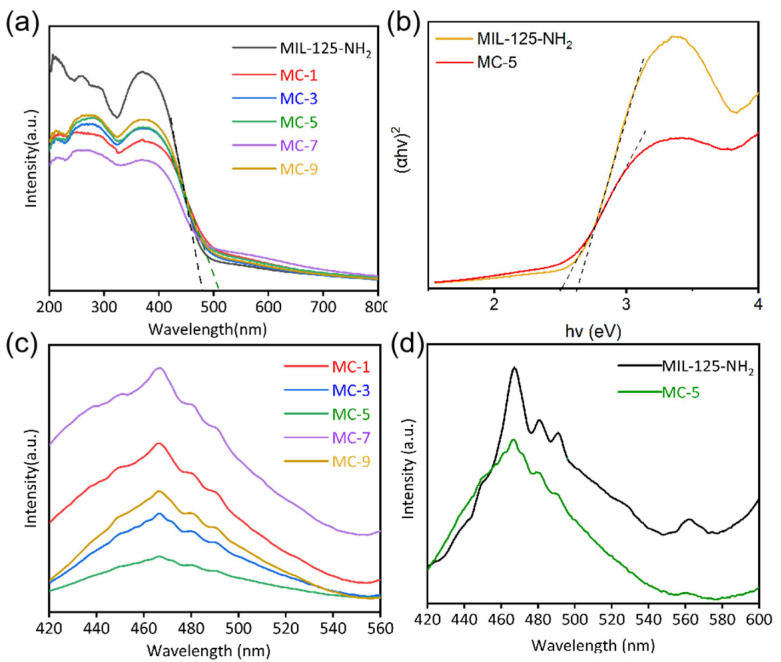
(**a**) UV–vis diffuse reflectance spectra of different samples, (**b**) (αhν)^2^ versus hν curves of MIL-125-NH_2_ and MC-5, (**c**,**d**) PL spectra of different samples.

The optical characteristics of the various samples were examined using UV–vis diffuse reflectance spectroscopy. Figure 6a illustrates the absorption bands corresponding to the Ti-oxo-clusters within the 200–350 nm range. The presence of absorption edges at longer wavelengths in the composite aerogel signified enhanced absorption in the visible region, promoting the generation of electron–hole pairs and subsequently improving the photocatalytic activity of the materials [42]. The bandgap energy was calculated by extrapolating the straight portion of (*αhv*)^2^ versus *hv* according to the Kubelka–Munk method [18]. As evident from Figure 6b, the bandgap energy (Eg) of MIL-125-NH_2_ was 2.6 eV, and the Eg value of MC-5 was approximately 2.5 eV. The presence of cellulose has a weak influence on the bandgap of the MOFs. A narrow bandgap facilitates the formation of photogenerated electron–hole pairs, thereby enhancing photocatalytic efficiency. The recombination of electrons and holes results in photoluminescence emission. To examine charge carrier trapping and recombination, the photoluminescence spectra of MIL-125-NH_2_ and composite aerogels were obtained (Figure 6c,d). All samples exhibited a maximum emission at 467 nm, attributed to the π–π* transition of the benzene ring [43]. The PL spectra of the composites were comparable to those of the pristine MIL-125-NH_2_. Notably, MC-5 demonstrated superior electron–hole pair separation, as evidenced by its lowest PL intensity, aligning with the experimental finding of achieving the highest photocatalytic performance [44].

**Figure 7:**
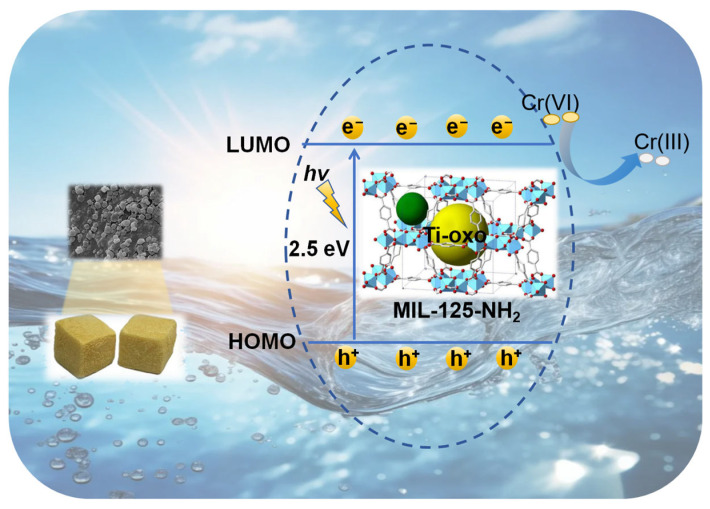
The proposed photocatalytic mechanism for Cr(VI) reduction with the composite aerogel.

Integrating the characterization results and experimental data, Figure 7 depicts a plausible mechanism for the photocatalytic reduction of Cr(VI) with the composite aerogel under visible-light irradiation. The electrostatic attraction arising from the opposing surface zeta potentials of the CNFs and MIL-125-NH_2_ facilitated their self-assembly into the composite material. Additionally, the non-covalent interactions between the MIL-125-NH_2_ and nanocellulose promoted interface affinity and MOF dispersion [45]. Consequently, the composite aerogels exhibited the excellent optical properties inherited from the catalyst, displaying robust responsiveness to visible light. As a porous material, the cellulose aerogel has a large specific surface area and a porous structure, which facilitate the transport of pollutants and light scattering [46]. When wastewater containing Cr(VI) permeates a composite aerogel via capillary action, the uniformly dispersed photocatalyst efficiently reduces Cr(VI) under visible light. The literature indicates that MIL-125-NH_2_ possesses a conduction band (LUMO) of −0.72 eV vs. NHE, which is notably lower than the Cr(VI)/Cr(III) reoxidation potential (1.33 eV vs. NHE at pH 2). Moreover, the photoexcited electrons generated by the organic ligand in MIL-125-NH_2_ are transferred to the Ti-O clusters, leading to the reduction of Ti^4+^ to Ti^3+^, which possesses potent reducing capabilities.

## 4. Conclusions

In summary, a shapeable composite aerogel featuring a porous structure was developed for the photocatalytic reduction of Cr(VI) in wastewater. This aerogel integrates MIL-125-NH_2_ within a carboxylated CNF matrix through a self-assembly approach. The hierarchical pore structure, created by electrostatic and acid–base interactions, exhibits excellent interfacial compatibility. The remarkable accessibility of the MOFs and the effective preservation of the microporous structure contribute to the efficient removal of Cr(VI), with an optimal removal efficiency of 99.8% achieved by the MC-5 sample. This composite aerogel holds potential for the remediation of heavy metal contamination.

## Data Availability

All data are included in this manuscript.

## Supplementary Materials

The following supporting information can be downloaded at: https://www.mdpi.com/article/10.3390/polym16223162/s1, Figure S1. The standard plot for determining Cr(VI) concentration; Figure S2. Zeta potential of MIL-125-NH_2_ suspensions at different pH values; Figure S3. (a) XRD patterns of fresh and recovered MC-5 aerogel after use, (b) SEM images of MC-5 composite foams before use, (c) SEM images of MC-5 composite foams after use; Table S1. BET parameters of all samples; Table S2. Comparison of Cr(VI) removal of similar MOF-based hybrid photocatalysts.
