# Supplementary material for: Amino-Functionalized Metal–Organic Framework-Mediated Cellulose Aerogels for Efficient Cr(VI) Reduction
Source: Polymers (Basel). 2024 Nov 13;16(22):3162. doi: 10.3390/polym16223162 (PMC11598302; doi:10.3390/polym16223162)
Supplement: Supplementary file 1 [file polymers-16-03162-s001.zip › polymers-3292765-supplementary.pdf]

Supporting information

for

The procedures for Cr(VI) detection (diphenyl carbazide method):

(1) Use a pipette to transfer 0, 2.0, 4.0, 6.0, 8.0, and 10.0 mL of Cr(VI) standard solution into six 50 mL colorimetric tubes respectively, and dilute them to the mark with water.

(2) Add sulfuric acid solution (0.2 mol L^{-1}) to adjust the pH of the solution to 1-3.

(3) Add 2 mL of diphenylcarbazide into the solution under shaking. After 5 to 10 minutes, measure the absorbance of each standard solution at a wavelength of 540 nm using water as a reference. Plot the standard curve with the concentration of Cr(VI) ions as the horizontal axis and the absorbance as the vertical axis.

(4) Determine the Cr(VI) concentration in the polluted samples by comparing with the standard curve. Measure and record the absorbance of each standard solution at 540 nm, and calculate the specific concentration of Cr^{6+} (mg/L) using the formula m/V.

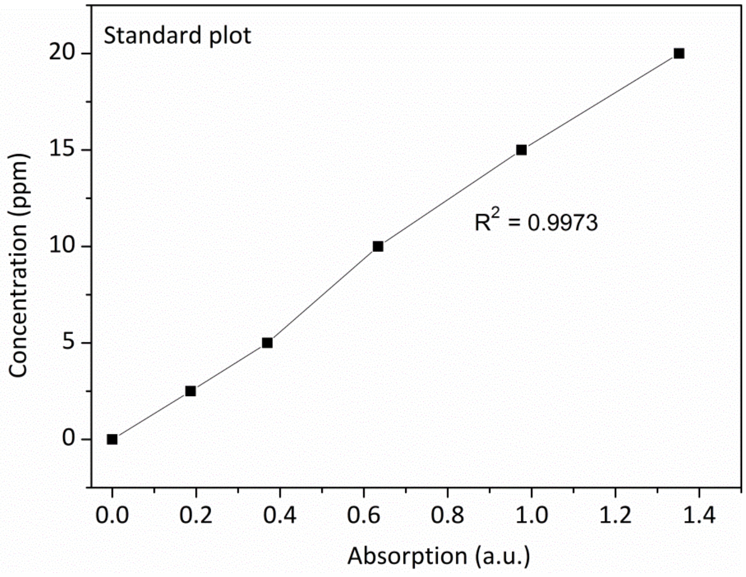

Figure S1. The standard plot for determining the C/C_0

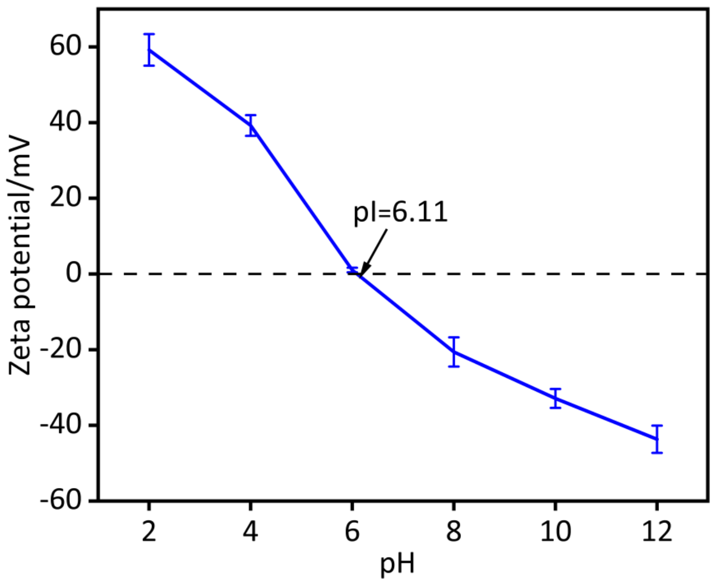

Figure S2. Zeta potential of MIL-125-NH_2 suspensions at different pH

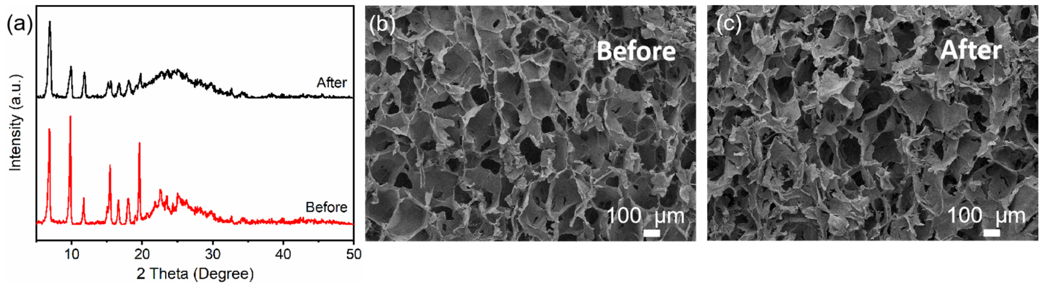

Figure S3. a) XRD patterns of fresh and recovered MC-5 aerogel after use, b) SEM images of MC-5 composite foams before use, c) SEM images of MC-5 composite foams after use.

Table S1. The BET parameters of different samples

Samples	BET surface area (m^2/g)	t-Plot micropore area (m^2/g)	Pore volume (cm^3/g)
MIL-125-NH_2	932	889	0.56
Cellulose aerogel (C-A)	74	-	0.11
Hybrid aerogel (MC-5)	582	525	0.38

Table S2. Comparison of similar MOFs based hybrid photocatalysts in Cr(VI) removal

Samples	Dosage (mg)	Concentration (mg/L)	Degradation efficiency (%)	Year
MIL-101-NH_2@cellulose foam	80	6	100	2021[S1]
PAN/CA/MIL-125/TiO_2-20%	100	50	100	2020[S2]
MIL-100(Fe)/cellulose/PVP	15	10	38	2021[S3]
5% polypyrrole nanotube@NH_2-MIL-125	50	10	99.02	2022[S4]
UIO-66/polyacrylonitrile	30	10	93	2022[S5]
MC-5	40	10	99.8	This work

References

[S1] Liu, J.; Hao, D.D.; Sun, H.W.;et al. Integration of MIL-101-NH_2 into cellulosic foams for efficient Cr (VI) reduction under visible light. *Ind. Eng. Chem. Res.*, **2021**. 60(33), 12220-12227.

[S2] Bahmani, E.; Seyyed, Z.H.; Koushkbaghi, S.; et al. Electrospun polyacrylonitrile/cellulose acetate/MIL-125/TiO2 composite nanofibers as an efficient photocatalyst and anticancer drug delivery system. *Cellulose*, **2020**. 27(17), 10029-10045.

[S3] Lu, W.L.; Duan, C.; Zhang, Y.; et al. Cellulose-based electrospun nanofiber membrane with core-sheath structure and robust photocatalytic activity for simultaneous and efficient oil emulsions separation, dye degradation and Cr(VI) reduction. *Carbohyd. Polym.* **2021**. 258, 117676.

[S4] Choe, J.N.; Yang, X.; Yu, J.H.; et al. Visible- light responsive PPynt@NH_2-MIL-125 nanocomposite for efficient reduction of Cr(VI). *Colloids Surf. A.* **2022**. 637, 128147.

[S5] Zhou, M.M.; Zou, W.; Zhu, X.M.; et al. In situ growth of UIO-66-NH_2 on thermally stabilized electrospun polyacrylonitrile nanofibers for visible-light driven Cr (VI) photocatalytic reduction. *J. Solid State Chem.*, **2022**. 307, 122836.
